# Ginseng Rh2 protects endometrial cells from oxygen glucose deprivation/re-oxygenation

**DOI:** 10.18632/oncotarget.22390

**Published:** 2017-11-11

**Authors:** Xiao-Fang Tang, Hai-Yan Liu, Ling Wu, Min-Hui Li, Shu-Ping Li, Hong-Bin Xu

**Affiliations:** ^1^ Obstetrics and Gynecology Department, Changzhou Second People's Hospital, Changzhou, China; ^2^ Obstetrics and Gynecology Department, Maternal and Child Health Care Hospital of Yancheng City, Yancheng, China

**Keywords:** Ginseng Rh2, oxygen glucose deprivation/re-oxygenation, endometrial cells, programmed necrosis, cyclophilin

## Abstract

In this study, oxygen glucose deprivation/re-oxygenation (OGDR) was applied to cultured endometrial cells to mimic ischemic-reperfusion injuries. We also tested the potential effect of Ginseng Rh2 (GRh2) against the process. In established T-HESC human endometrial cells and primary murine endometrial cells, GRh2 largely inhibited OGDR-induced viability reduction and cell death. Remarkably, OGDR induced programmed necrosis in the endometrial cells, evidenced by cyclophilin D-p53-adenine nucleotide translocator 1 (ANT-1) mitochondrial association, mitochondrial depolarization, reactive oxygen species production, and lactate dehydrogenase release. Notably, such effects by OGDR were largely attenuated with co-treatment of GRh2. Further, cyclophilin D inhibition or knockdown also protected endometrial cells from OGDR. On the other hand, forced over-expression of cyclophilin D facilitated OGDR-induced T-HESC cell necrosis, which was dramatically inhibited by GRh2. Together, GRh2 protects endometrial cells from OGDR possibly via inhibiting CypD-dependent programmed necrosis pathway.

## INTRODUCTION

Postpartum hemorrhage is one common complication in obstetrics [1–3], which will cause ischemic damages to the endometrium [1–3]. In the clinical practice, endometrium ischemia is often followed by reperfusion, which shall lead to profound oxidative damages to the surrounding endometrial cells and tissues [1–3]. Ischemia-reperfusion is known to induce profound reactive oxygen species (ROS) production [4–6]. In addition, sustained oxidative stress shall also cause increased level of circulating lipid peroxides, along with concomitant reduction of the antioxidants [4–6]. These together could cause further injuries to the endometrial cells/tissues [1–3].

Oxygen and glucose deprivation (OGD) and following re-oxygenation (OGDR) is applied to the cultured cells, mimicking the pathological condition of ischemia-reperfusion injuries [7–10]. Interestingly, recent evidences have suggested that OGDR could induce cell necrosis, (but not apoptosis) [7, 10]. The conventional view is that cell necrosis is a passive form of cell death. Yet, recent studies have implied that necrosis could also be active, programmed and energy-consuming [11–14]. The “programmed necrosis” is mitochondrial dependent [15], which mediates cell death by a number of stimuli, including oxidative stress, calcium over-load, ultra-violet radiation (“UVR”), and several chemo-agents [11, 12, 16, 17]. Studies have shown that many different stimuli could induce p53 translocation to cell mitochondria, where it forms a complex with mitochondria permeability transition pore (mPTP) structural protein cyclophilin-D (“CypD”). The complexation shall dictate mitochondrial depolarization, mPTP opening, cytochrome C release and ROS production. This would eventually induce cell necrosis (but not apoptosis) [11–13, 16–19]. One goal of this study is to test whether this necrosis pathway also participates OGDR-induced endometrial cell death *in vitro*.

Ginsenosides are the primary active constituents in the root of Ginseng [20, 21]. Ginsenoside Rh2 (GRh2) is a converted ginsenoside, which has shown various pharmaceutics activities [20, 21]. In the current study, we show that GRh2 protects endometrial cells from OGDR possibly via inhibiting programmed necrosis pathway.

## RESULTS

### GRh2 protects endometrial cells from OGDR

**Figure 1:**
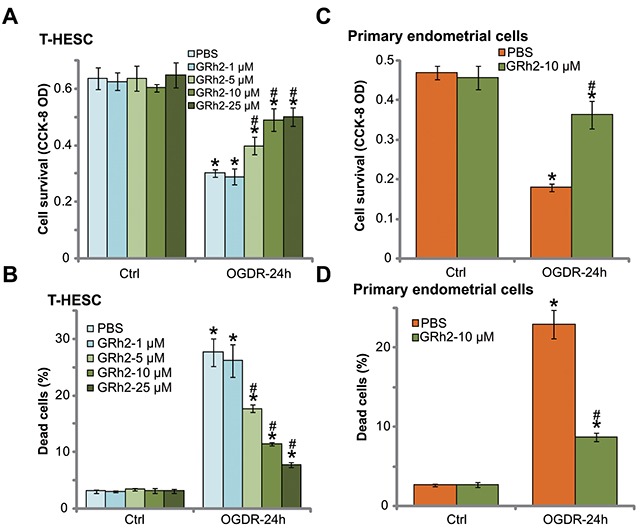
GRh2 protects endometrial cells from OGDR The T-HESC human endometrial cells **(A** and **B)** or the primary murine endometrial cells **(C** and **D)** were treated with applied concentration (1-25 μM) of GRh2, together with/out OGD exposure for 4 hours, followed by 24 hours of re-oxygenation (“OGDR”), cell survival was tested by CCK-8 assay (A and C); cell death was examined by the trypan blue staining assay (B and D). “OGDR” stands for OGD/re-oxygenation (same for all figures). “Ctrl” stands for untreated control cells (same for all figures). For the OGDR experiments, endometrial cells were always pre-treated with applied concentration of GRh2 for 30 min before OGD (same for all figures). Bars stands for mean ± standard deviation (SD, n=5). ^*^ *p*<0.05 *vs.* “Ctrl”. ^#^ *p*<0.05 *vs.* cells with “OGDR” only treatment (no GRh2). Each experiment was repeated four times with similar results obtained.

The current study aimed to test the potential effect of Ginseng Rh2 (GRh2) on OGD/re-oxygenation (OGDR)-treated endometrial cells. First, T-HESC cells, an established human endometrial cell line [22], were treated with gradually-increased concentrations of GRh2, from 1-25 μM. The cell counting kit-8 (CCK-8) assay results in Figure 1A demonstrated that treatment of GRh2 alone at tested concentrations (1-25 μM, for 24 hours) didn't change the survival of T-HESC endometrial cells. Meanwhile, GRh2 treatment also failed to induce T-HESC cell death, the latter was tested by trypan blue staining assay (Figure 1B). Notably, exposure of T-HESC cells with OGD (for 4 hours)/re-oxygenation (“OGDR”, for another 24 hours) induced potent viability reduction (CCK-8 OD decrease, Figure 1A) and cell death (trypan blue staining increase, Figure 1B). Remarkably, such effects by OGDR were largely attenuated with co-treatment of GRh2 (Figure 1A and 1B). GRh2, at 5-25 μM, significantly inhibited OGDR-induced T-HESC cell viability reduction (Figure 1A) and cell death (Figure 1B). Notably, GRh2-induced T-HESC cell protection against OGDR was dose-dependent (Figure 1A and 1B). At a low concentration (1 μM), GRh2 was however ineffective (Figure 1A and 1B).

The potential effect of GRh2 in the primary endometrial cells was also tested. As demonstrated, treatment the primary murine endometrial cells with the same OGDR procedure (4 hours of ODG plus 24 hours of re-oxygenation) induced profound viability (CCK-8 OD) reduction (Figure 1C) and cell death (Figure 1D). Co-treatment with 10 μM of GRh2 again significantly attenuated OGDR-induced cytotoxicity in the primary murine endometrial cells (Figure 1C and 1D). GRh2 alone was non-cytotoxic to the primary endometrial cells (Figure 1C and 1D). Together, these results suggest that GRh2 protects endometrial cells from OGDR.

### OGDR fails to induce endometrial cell apoptosis

**Figure 2:**
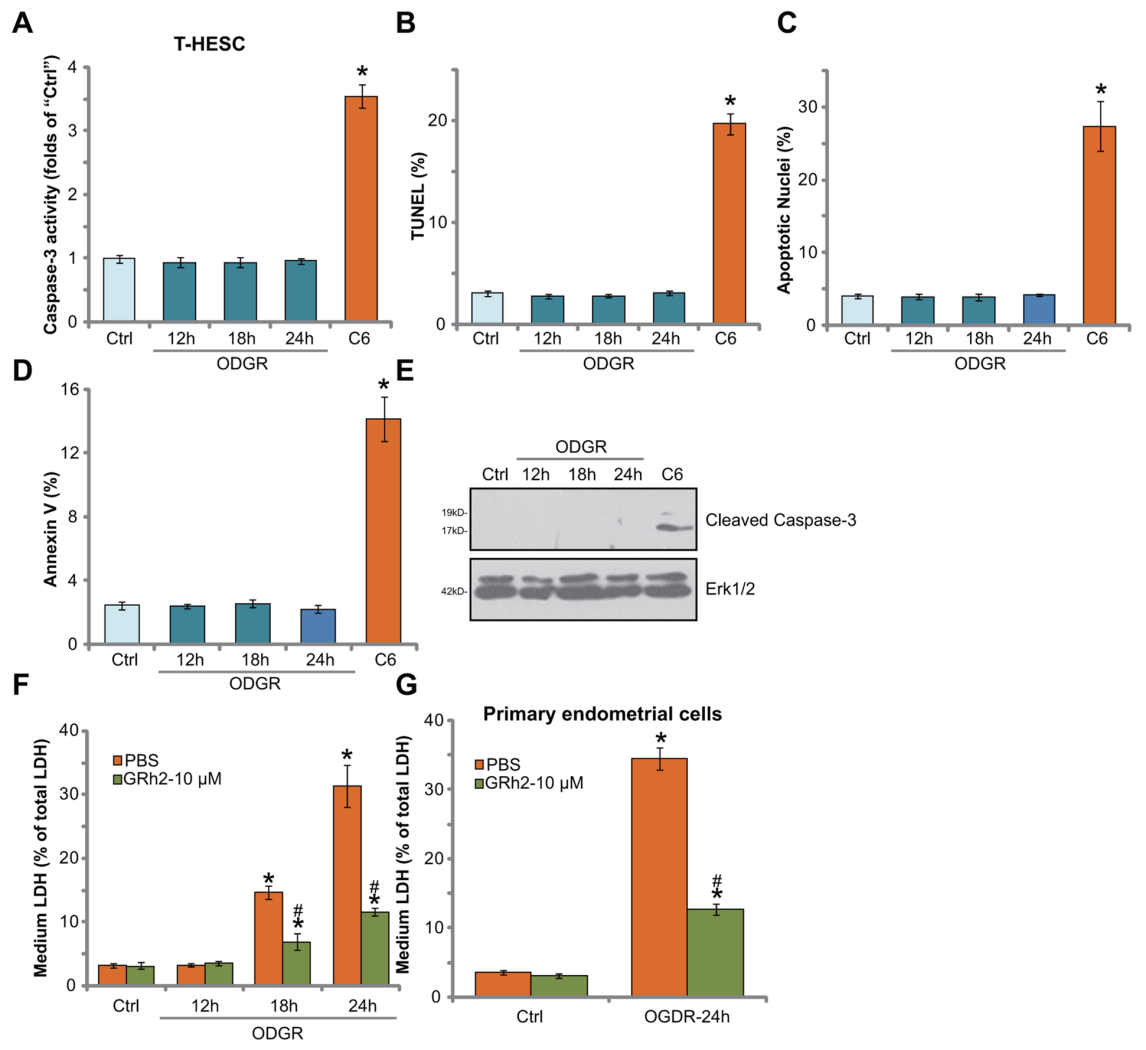
OGDR fails to induce endometrial cell apoptosis The T-HESC human endometrial cells **(A-F)** or the primary murine endometrial cells **(G)** were treated with GRh2 (10 μM), with/out OGDR exposure, after applied time, cell apoptosis was tested by the assays mentioned in the text (A-E). LDH release in the conditional medium was tested as the indicator of cell necrosis (F and G). For testing cell apoptosis, cell permeable short-chain C6 ceramide (“C6”, 20 μM, 24 hours) was added as the positive control (A-E). Bars stands for mean ± standard deviation (SD, n=5). ^*^ *p*<0.05 *vs.* “Ctrl”. ^#^ *p*<0.05 *vs.* cells with “OGDR” only treatment (no GRh2). Each experiment was repeated four times with similar results obtained.

Cell apoptosis is a main reason of cell death in response to different stimuli. We thus tested possible apoptosis induction in OGDR-treated endometrial cells. Various established apoptosis assays were performed, including the caspase-3 activity assay (Figure 2A), TUNEL nuclei staining assay (Figure 2B), Hoechst33342-apoptotic nuclei assay (Figure 2C), Annexin V FACS assay (Figure 2D) and Western blotting assay of cleaved-caspase-3 (Figure 2E). Intriguingly, OGDR exposure failed to induce significant apoptosis in T-HESC endometrial cells (Figure 2A-2D). OGDR exposure, for different time points (12, 18 and 24 hours), didn't induce notable increase in the caspase-3 activity (Figure 2A), percentage of apoptotic nuclei (Figure 2B and 2C), nor the Annexin V percentage (Figure 2D). Further, ODGR failed to induce caspase-3 cleavage in T-HESC cells (Figure 2E). On the other hand, the short-chain C6 ceramide, a well known apoptosis inducer [23–25], provoked significant apoptosis activation in T-HESC cells (Figure 2A-2E).

Cell necrosis is another form of cell death [15]. Lactate dehydrogenase (LDH) release to the medium is often detected as a marker of cell necrosis *in vitro*. In the current study, we showed that medium LDH content was significantly increased following OGDR exposure in T-HESC endometrial cells (Figure 2F). Remarkably, such effect was largely inhibited by co-treatment of GRh2 (Figure 2F). These results imply that GRh2 might protect T-HESC cells from OGDR via inhibiting cell necrosis, but not apoptosis. In the primary murine endometrial cells, GRh2 (10 μM) similarly inhibited OGDR-induced cell necrosis (LDH release, Figure 2G).

### GRh2 prevents OGDR-induced programmed necrosis in endometrial cells

**Figure 3:**
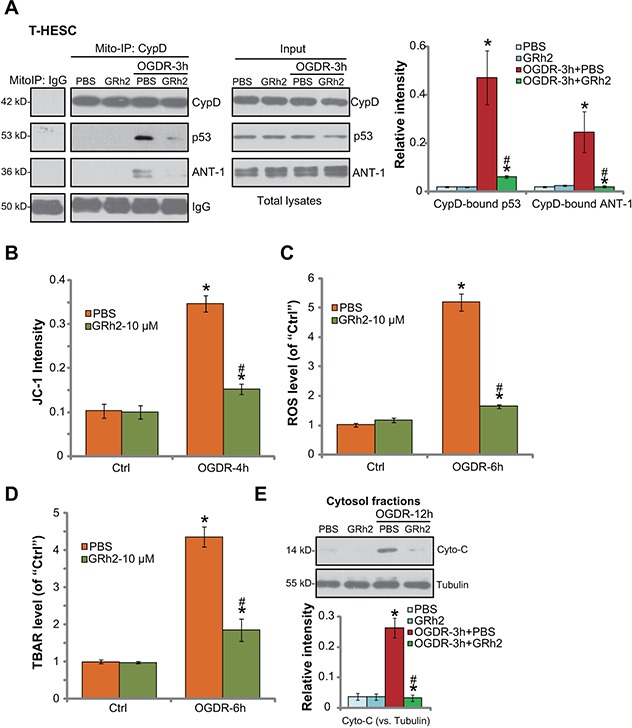
GRh2 prevents OGDR-induced programmed necrosis in endometrial cells T-HESC human endometrial cells were treated with GRh2 (10 μM), together with/out OGDR exposure, after applied time, mitochondrial CypD-p53-ANT-1 association (“Mito-IP”, **A**, “Input” showed expression of the proteins in total cell lysates), mitochondrial depolarization (JC-1 intensity OD, **B**), ROS production **(C)**, lipid peroxidation **(D)** and cytochrome C release (**E**, testing cytosol proteins) were tested by the assays mentioned in the text. For the Mito-IP assay, CypD-bound p53 and CypD-bound ANT-1 were quantified (A, the right panel). For the cytochrome C release assay, relative cytosol cytochrome C level (*vs.* Tubulin) was also quantified (E, the lower panel). Bars stands for mean ± standard deviation (SD, n=5). ^*^ *p*<0.05 *vs.* “Ctrl”. ^#^ *p*<0.05 *vs.* cells with “OGDR” only treatment (no GRh2). Each experiment was repeated three times with similar results obtained.

As discussed, a number of stimuli and stresses could activate the programmed necrosis pathway to promote cell death [11–13, 16–19]. We wanted to know if this pathway was also induced in OGDR-treated endometrial cells. The mitochondrial immunoprecipitation (“Mito-IP”) assay [12, 26, 27] results in Figure 3A showed that, following OGDR exposure, the mPTP structure protein CypD formed a complex with p53 and ANT-1 (adenine nucleotide translocator 1), the latter is another mPTP component protein [16, 28–30]. Expressions of CypD, p53 and ANT-1 were unchanged after OGDR (Figure 3A, “Input”). OGDR-induced CypD-p53-ANT-1 mitochondrial association was followed by mitochondrial depolarization (JC-1 green fluorescence intensity increase, Figure 3B) and ROS production (H2DCF-DA fluorescence intensity increase, Figure 3C), as well as lipid peroxidation (TBAR activity) increase (Figure 3D) and cytochrome C cytosol release (Figure 3E). All these evidences clearly indicated induction of programmed necrosis (see previous studies [11–13, 16–19]) by OGDR in endometrial cells. Remarkably, such effects by OGDR were largely attenuated with co-treatment of GRh2 (Figure 3A-3E). More specifically, GRh2 potently inhibited OGDR-induced mitochondrial CypD-p53-ANT-1 association (Figure 3A, also see the quantification results), mitochondrial depolarization (Figure 3B), oxidative stresses (Figure 3C and 3D) and cytochrome C release (Figure 3E, also see the quantification results). GRh2 alone, as expected, had no effect on the programmed necrosis pathway activation (Figure 3A-3E). Together, our results suggest that GRh2 prevents OGDR-induced programmed necrosis pathway activation in endometrial cells.

### Inhibition of CypD prevents OGDR-induced endometrial cell programmed necrosis

**Figure 4:**
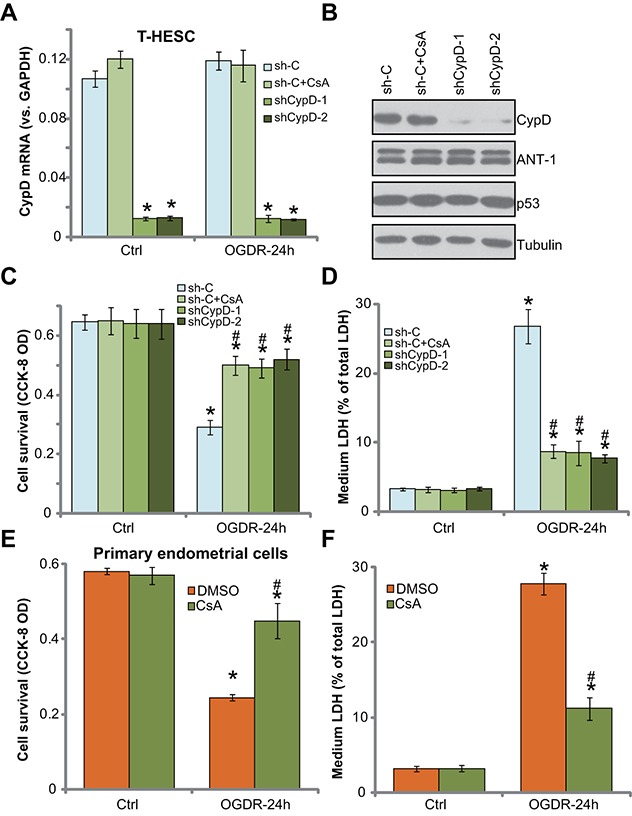
Inhibition of CypD prevents OGDR-induced endometrial cell programmed necrosis T-HESC cells, expressing the scramble non-sense control shRNA (“sh-C”) or CypD-targeting shRNA (“shCypD”) were treated with/out OGD for 4 hours (“sh-C” cells were also co-treated with cyclosporin A “CsA,10 μM”), followed re-oxygenation (“OGDR”) for applied time, expressions of CypD and other listed genes were tested by qRT-PCR assay **(A)** and Western blotting assay **(B)**; cell survival and necrosis were tested by CCK-8 assay **(C)** and LDH release assay **(D)**, respectively. The primary murine endometrial cells were treated with 10 μM of CsA, together with/out OGDR for 24 hours, cell survival **(E)** and necrosis **(F)** were tested. Bars stands for mean ± standard deviation (SD, n=5). “DMSO” stands for 0.1% of DMSO. ^*^ *p*<0.05 *vs.* “Ctrl”. ^#^ *p*<0.05 *vs.* cells with “OGDR” only treatment (no GRh2). Each experiment was repeated three times with similar results obtained.

To further confirm that programmed necrosis pathway activation is required for OGDR-induced endometrial cell death, pharmacologic and shRNA methods were employed to inhibit CypD. Cyclosporin A (CsA) is a known CypD inhibitor, which is shown to shut down the programmed necrosis pathway [11, 12, 31, 32]. Additionally, two shRNAs, against distinct and non-overlapping sequence of CypD (a gift from Dr. Guo [11]), were applied. *CypD mRNA* (Figure 4A) and protein (Figure 4B) expressions were largely downregulated in the stable T-HESC cells with the CypD shRNA. CsA or OGDR alone didn't change CypD expression (Figure 4A). As demonstrated, inhibition of CypD by CsA or the two targeted-shRNAs largely attenuated OGDR-induced T-HESC cell viability (CCK-8 OD) reduction (Figure 4C) and cell necrosis (LDH medium release, Figure 4D). Thus, inhibition of CypD activation (by CsA) or expression (by targeted-shRNA) could efficiently alleviate OGDR-induced T-HESC cell death (Figure 4C and 4D). These evidences further suggest that CypD-dependent necrosis pathway mediates cell death by OGDR. In the primary murine endometrial cells, the CypD inhibitor CsA similarly alleviated cell death (CCK-8 OD reduction, Figure 4E) and necrosis (LDH release, Figure 4F) by OGDR. It should be noted that CypD inhibition or knockdown alone failed to change endometrial cell survival and death (Figure 4A-4F).

### Forced over-expression of CypD facilitates OGDR-induced endometrial cell death, inhibited by GRh2

**Figure 5:**
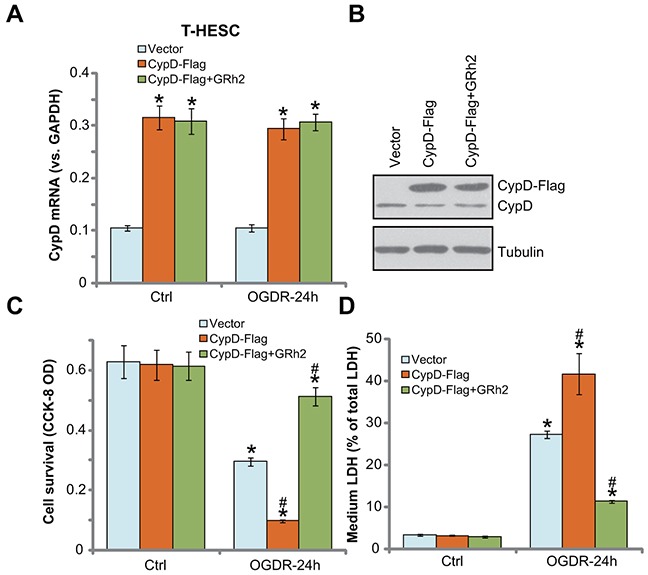
Forced over-expression of CypD facilitates OGDR-induced endometrial cell death, inhibited by GRh2 T-HESC cells, expressing the empty vector (pSuper-puro-Flag, “Vector”) or CypD-Flag vector (“CypD-Flag”), were treated with GRh2 (10 μM), together with/out OGDR exposure for 24 hours, *CypD mRNA* and protein expressions were tested by qRT-PCR assay **(A)** and Western blotting assay **(B)**, respectively; cell survival and necrosis were tested by CCK-8 assay **(C)** and LDH release assay **(D)**, respectively. Bars stands for mean ± standard deviation (SD, n=5). ^*^ *p*<0.05 *vs.* “Ctrl”. ^#^ *p*<0.05 *vs.* cells with “OGDR” only treatment (no GRh2). Each experiment was repeated three times with similar results obtained.

Based on the above results, we propose that CypD-dependent programmed necrosis pathway activation mediates OGDR-induced endometrial cell death. We therefore speculated that forced over-expression of CypD might possibly facilitate OGDR-induced endometrial cell death. To test this hypothesis, a CypD-expression vector (a gift from Dr. Fang [13]) was applied. The exogenous CypD construct was tagged with Flag, and was transfected to T-HESC endometrial cells. *CypD mRNA* expression was significantly increased in cells with the construct (Figure 5A), which was not changed by GRh2 nor OGDR (Figure 5A). Western blotting assay results in Figure 5B further confirmed the expression of exogenous CypD (Flag-tagged) in the stable cells. Significantly, OGDR-induced viability reduction (Figure 5C) and cell necrosis (Figure 5D) were significantly boosted in T-HESC cells with the exogenous CypD. These results demonstrated that over-expression of CypD indeed facilitated OGDR-induced endometrial cell death. Notably, co-treatment with GRh2 potently inhibited OGDR-induced death even in the CypD-over-expressed T-HESC cells (Figure 5C and 5D). These results again suggest that GRh2-induced endometrial cell protection is possibly through inhibiting CypD-dependent programmed necrosis pathway.

## DISCUSSION

p53 is a key molecule to mediate cell apoptosis [33–37]. Recent studies, however, have implied that p53 is also required for the induction of cell necrosis. Various stimuli and stresses, *i.e*. hypoxia, calcium overload, and oxidative stresses, could trigger p53 translocation to mitochondria, where it associates with CypD [27]. Mitochondrial Cyp-D-p53 complexation is proven to be required for the mitochondrial depolarization [27], and more importantly, subsequent cell necrosis (programmed necrosis). Inhibition of the CypD-p53 complex, genetically or pharmacologically, could efficiently protect cells from the stresses [27].

In the current study, we proposed that OGDR also induced programmed necrosis in endometrial cells, which was evidenced by mitochondrial CypD-p53-ANT-1 association (a key step to initiate necrosis [11, 12, 17, 26]), mitochondrial depolarization, ROS production, and LDH medium release (the end point). On the other hand, inhibition of programmed necrosis pathway, by CypD shRNA or its inhibitor CsA, significantly inhibited OGDR-induced endometrial cell death. Notably, results of various apoptosis assays indicated that OGDR failed to induce significant apoptosis activation in endometrial cells. Therefore, CypD-dependent programmed necrosis pathway could be an important therapeutic target for the treatment of endometrial cell OGDR/ischemia-reperfusion injuries.

Although GRh2 has displayed many different functions in various cell types, this is, as far as we know, the first study to test its potential activity in endometrial cells. Here, we showed that GRh2 efficiently protected T-HESC human endometrial cells and primary murine endometrial cells from OGDR. At the molecular level, we propose that inhibition of the programmed necrosis pathway could be the primary mechanism of its cytoprotection against OGDR. Although the underlying mechanisms need further characterizations, our results imply that it would be interesting to further test GRh2′s activity against endometrial cell ischemia-reperfusion injuries.

## METERIALS AND METHODS

### Chemical and reagents

GRh2, the CypD inhibitor cyclosporine A (CsA), and puromycin were purchased from Sigma-Aldrich (Shanghai, China). The two non-overlapping lentiviral CypD shRNAs (“-1/−2”) as well as the scramble control non-sense lentiviral shRNA were provided by Dr. Guo [11]. All the reagents for cell culture were purchased from Gibco (Suzhou, China). GRh2 was always freshly dissolved in warm phosphate buffer solution (PBS) before the experimental usage. The antibodies utilized in this study were provided by Santa Cruz Biotechnology (Santa Cruz, CA) and Cell Signaling Tech (Suzhou, China). The short-chain C6 ceramide was purchased from Avanti Polar Lipids (Alabaster, Alabama).

### Culture T-HESC human endometrial cells

The immortalized human endometrial cell line, T-HESC [22], was purchased from the Cell Bank of Shanghai Institute of Biological Science of CAS (Shanghai, China). The T-HESC cells were cultured in DMEM-F12 medium with 10% fetal bovine serum (FBS), and 4 mM L-glutamine, 0.25% HEPES plus necessary antibiotics, at 37°C in a humidified atmosphere of 5% CO_2_ [22].

### The primary culture of murine endometrial (stromal) cells

Female, 8-12 week-old C57BL/6J mice were purchased from the Experimental Animal Center of Soochow University (Suzhou, China). Animals were housed in filter-top cages under conventional conditions. The detailed protocol was described in detail in a previous study [38]. Three days prior to hysterectomy, mice (n = 5/culture) were injected *s.c*. with 100 ng of estrogen E2 solution to synchronize the cycle. The acquired primary mouse uterine tissues were then incubated with 0.05% trypsin-EDTA supplemented with 1 mg/mL collagenase (Sigma-Aldrich) for 30 min at 37°C. Next, the uterine tissues were transferred to DMEM/Hams F-12 nutrient plus 10% FBS. After 5 min, tissues were transferred into 3 mL of ice-cold HBSS and vortexed. The resulting epithelial cells were abandoned using gravity sedimentation, and the step was repeated three times. Afterwards, the primary endometrial stromal cells were pelleted and resuspended in the complete DMEM medium with 10% FBS (Gibco, Belgium), 0.5 mg/mL fungizone and 100 mg/mL gentamicin. The animal protocol was approved by the Ethics Review Board of Soochow University (Suzhou, China).

### Cell viability assay

The viability of endometrial cells was tested by cell counting kit-8 (CCK-8) kit (Dojindo Laboratories, Kumamoto, Japan), according to the standard procedure. The CCK-8 optic density (OD) at 450 nm was recorded.

### Trypan blue staining of “dead” cells

Dead cells were positively stained with trypan blue dye (Sigma), and the percentage (%) of trypan blue-stained cells was recorded (using an automatic cell counter), indicating cell death ratio.

### Lactate dehydrogenase (LDH) assay

The release of LDH from the intact cells to the conditional medium is a characteristic marker of cell necrosis *in vitro* [39]. In this study, a two-step LDH detection kit (Promega, Shanghai, China) was employed to test LDH level in the medium, and its level was always normalized to total LDH (combination of medium LDH and cellular LDH).

### OGD/re-oxygenation (OGDR)

The OGDR procedure was described in a previous study [40]. Briefly, endometrial cells were first placed into an airtight chamber and equilibrated for 10 min with a continuous flux of gas (95% N_2_/5% CO_2_). The chamber was sealed and placed in an incubator for 4 hours (mimic oxygen glucose deprivation or OGD). Afterwards, the endometrial cells returned back to the complete medium and re-oxygenated. Control cells were placed in norm-oxygenated complete medium.

### Western blotting assay

Following the indicated treatment, the endometrial cells were incubated with RIPA lysis buffer (Biyuntian, Wuxi, China). The quantified protein lysate samples (40 μg per treatment of each lane) were separated by the 10-12% SDS-PAGE gels, and were transferred to the polyvinylidene difluoride (PVDF) membrane [41]. The detailed protocol of Western blotting assay and data quantification were described previously [42, 43].

### Caspase-3 activity assay

For each treatment, 20 μg of cytosolic extracts were incubated with the caspase assay buffer [12] together with the caspase-3 substrate [12] for 2 hours at the room temperature, the release of 7-amido-4-(trifluoromethyl) coumarin (AFC) was quantified, using a Fluoroskan system set [12]. The AFC's OD value (at 405 nm) was recorded to reflect caspase-3 activity.

### Hoechst-33342 nuclei staining of apoptotic cells

Cells were stained with Hoechst-33342 (Sigma). Non-apoptotic nuclei were with faint delicate chromatin staining, but nuclei with intensified Hoechst-33342 condensation/brightness (early apoptotic cells) or fragmentation (late apoptotic cells) were labeled as apoptotic cells. Apoptotic nuclei percentage was calculated, from at least 300 cells of 5 random views of each treatment.

### Other apoptosis assays

The Annexin V FACS assay of cell apoptosis and TUNEL nuclei staining assay of apoptosis were described in detail in the previous studies [44]. Annexin V ratio was sorted by Becton-Dickinson FACScan (Shanghai, China). TUNEL ratio (*vs.* total number of cells) was calculated, from at least 300 cells of 5 random views for each treatment.

### Mitochondrial immunoprecipitation (Mito-IP)

After the applied treatment, T-HESC cells were harvested and homogenized by the buffer of 250 mM sucrose, 20 mM HEPES, 10 mM KCl, 1.5 mM MgCl_2_, 1 mM EDTA, 1 mM EGTA, and 1 mM dithiothreitol. After centrifugation, the supernatants were collected as the cytosolic fractions. The resulting pellets were then re-suspended in the above buffer plus 10 μL NP-40, which were the mitochondrial fraction lysates. The quantified mitochondrial lysates (400 μg per sample) were pre-cleared and incubated with anti-CypD antibody [27, 45]. The mitochondrial complexes were then captured by the protein G-Sepharose beads (Sigma). CypD-p53-ANT-1 association was then tested by Western blotting assay.

### Mitochondrial depolarization assay

JC-1 fluorescent intensity change is often tested to reflect the mitochondrial membrane potential change [46]. With mitochondrial depolarization (“ΔΨ”), the red JC1 aggregates shall form the green monomers [47]. Testing mitochondrial depolarization by the JC-1 protocol was discussed in previous studies. Briefly, T-HESC cells were incubated with JC-1 (10 μg/mL, Invitrogen, Shanghai, China) for 10 min at the room temperature under the dark. JC-1 green fluorescence OD, reflecting mitochondrial depolarization, was examined immediately on a fluorescence spectrofluorometer at 530 nm (Titertek Fluoroscan, Germany).

### ROS detection

The fluorescent dye DCFH-DA (2′,7′-dichlorofluorescein diacetate) assay was applied to examine cellular ROS level [48–50]. DCFH-DA will be hydrolysed by intracellular esterases to DCF. Briefly, T-HESC cells with the treatment were incubated with DCFH-DA (100 μM, Invitrogen) for 60 min at the room temperature under the dark. The DCF fluorescence intensity at 530 nm, reflecting cellular ROS intensity, was detected by the above-described fluorescence reader (Titertek Fluoroscan, Germany).

### Lipid peroxidation assay

As previously described [50], cellular lipid peroxidation was evaluated by the thiobarbituric acid reactive substances (TBAR) assay [51]. Briefly, 20 μg lysate proteins of each treatment were first mixed with 20% of acetic acid and thiobarbituric acid solution. After heating, the mixture was centrifuged, and the red pigment dye in the supernatant was examined by the microplate reader [51]. TBAR activity was expressed as nM of malondialdehyde per mg protein.

### qRT-PCR

After treatment, cellular RNA was extracted by the RNeasy Mini Kit (Qiagen, Shanghai, China). Complementary DNA (cDNA) was synthesized from 1 μg of total RNA via a High Capacity cDNA Reverse Transcription kit (Applied Biosystems, Shanghai, China). The ABI Prism 7600 Fast Real-Time PCR system was utilized to perform the quantitative real time-PCR (qRT-PCR) assay. For each assay, melt curve analysis was performed to calculate product melting temperature. *Glyceraldehyde-3-phosphatedehydrogenase (GAPDH) mRNA* was tested as the reference gene, and the 2^−ΔΔ*C*t^ method was applied to quantify CypD mRNA expression change. The mRNA primers of human *CypD* and *GAPDH* were described previously [52].

### CypD shRNA

T-HESC cells were cultured at 50% confluence in low-serum (1%) medium in six-well tissue culture plate. The CypD shRNA lentivirus (10 μL/mL medium per well) or the scramble control shRNA lentivirus (10 μL/mL medium per well) was added to T-HESC cells overnight. Stable clones were selected with puromycin (1 μg/mL) starting at 24 hours after infection, for a total of 8 days. All the resulting stable cells were then assayed for knockdown of CypD via qRT-PCR assay and Western blotting assy.

### CypD over-expression

The CypD pSuper-puro-Flag vector and the empty vector were provided by Dr. Fang [13]. The CypD construct or the vector was transfected through Lipofectamine 2000 reagent (Invitrogen, Suzhou, China) to the cultured T-HESC cells. After 24 hours, cells were selected by puromycin (1.0 μg/mL) for another 8 days. CypD expression in the resulting stable cells was verified by both qRT-PCR assay and Western blotting assy.

### Statistical analysis

All data were presented as mean ± standard deviation (SD). Repeated-measures analysis of variance (RMANOVA) followed by Dunnett's post hoc test for multiple comparisons (SPSS 16.0) were applied to evaluate statistical significance of observed differences.

## CONCLUSION

Postpartum hemorrhage remains a prominent cause of maternal morbidity and mortality, which could possibly be managed by using a stepwise progressive approach [1–3]. Studies have demonstrated that manual and pharmacologic interventions are first-line treatments [1–3]. Our results provided new mechanistic insights (programmed necrosis) and related intervention strategies (GRh2) of ischemia-reperfusion-related endometrial cell injuries.
